# Supplementary material for: Wandering principal optical axes in van der Waals triclinic materials
Source: Nat Commun. 2024 Mar 6;15:1552. doi: 10.1038/s41467-024-45266-3 (PMC10918091; doi:10.1038/s41467-024-45266-3)
Supplement: Supplementary file 1 — Supplementary Information [file 41467_2024_45266_MOESM1_ESM.pdf]

Supplementary Information

Wandering principal optical axes in van der Waals triclinic materials

Georgy A. Ermolaev[1], Kirill V. Voronin[2], Adilet N. Toksumakov[3], Dmitriy V. Grudinin[1], Ilia M. Fradkin[1], Arslan Mazitov[4], Aleksandr S. Slavich[3], Mikhail K. Tatmyshevskiy[3], Dmitry I. Yakubovsky[3], Valentin R. Solovey[1], Roman V. Kirtaev[1], Sergey M. Novikov[3], Elena S. Zhukova[3], Ivan Kruglov[1], Andrey A. Vyshnevyy[1], Denis G. Baranov[3], Davit A. Ghazaryan[3,5], Aleksey V. Arsenin[1,5], Luis Martin-Moreno[6,7], Valentyn S. Volkov[1,5], and Kostya S. Novoselov[8,9,10,]*

[1]Emerging Technologies Research Center, XPANCEO, Dubai Investment Park First, Dubai, United Arab Emirates

[2]Donostia International Physics Center (DIPC), Donostia/San Sebastián 20018, Spain

[3]Moscow Center for Advanced Studies, Kulakova str. 20, Moscow, 123592, Russia

[4]Institute of Materials, École Polytechnique Fédérale de Lausanne, 1015 Lausanne, Switzerland

[5]Laboratory of Advanced Functional Materials, Yerevan State University, Yerevan 0025, Armenia

[6]Instituto de Nanociencia y Materiales de Aragón (INMA), CSIC-Universidad de Zaragoza, 50009 Zaragoza, Spain

[7]Departamento de Física de la Materia Condensada, Universidad de Zaragoza, 50009 Zaragoza, Spain

[8]National Graphene Institute (NGI), University of Manchester, Manchester, M13 9PL, UK

[9]Department of Materials Science and Engineering, National University of Singapore, Singapore, 03-09 EA, Singapore

[10]Chongqing 2D Materials Institute, 400714, Chongqing, China

*e-mail: kostya@nus.edu.sg

Table of Contents

Supplementary Note 1: Peculiarity of optical response of ReS_2.

Supplementary Note 2: Wavelength-dispersive principal optical axes in $ReSe_2$.

Supplementary Note 3: Raman spectra for ReS_2.

Supplementary Note 4: Static principal optical axes in As_2S_3.

Supplementary Note 5: Phenomenological non-orthogonal excitons model for wandering principal optical axes in ReS_2 and $ReSe_2$.

Supplementary Note 6: Angular dpendence of weak confinement modes in the reflection scheme of near-field microscopy.

Supplementary Note 7: Theoretical background of waveguide mode direction change – computational approach.

Supplementary Note 8: Theoretical background of waveguide mode direction change – near-field calculation analysis.

Supplementary Note 9: Comparison of calculated and measured near-fields.

Supplementary Note 10: Isotropic approximation for determination of ReS_2 optical constants.

Supplementary Note 11: First-principle calculations for $ReSe_2$ and monolayer, bilayer, and trilayer of ReS_2.

Supplementary Note 1: Peculiarity of optical response of ReS_2.

Materials with rotating optical axes have more degrees of freedom for optical response than those with fixed principal optical axes. As a result, their optical performance cannot be fully described by a dielectric function with static principal axes. At the same time, it might be possible to describe a particular optical measurement by an effective dielectric tensor having fixed principal axes. However, the obtained effective dielectric tensor might look unphysical or fail to describe other measurements. For example, work[1] extracts optical properties from near-field measurements (see Supplementary Figure 1a). From the Kramers–Kronig relations, one would expect the peak of optical absorption to appear for ε_y (see Supplementary figure 1b). We encounter a similar Kramers–Kronig "inconsistency" when we attempt to fit the unpolarized reflectance and transmittance spectra by isotropic dielectric function (see Supplementary Note 10 and Supplementary Figure 16f). In another paper[2], the authors find anisotropic optical properties by Mueller matrix ellipsometry. Yet, these constants are unable to explain our polarized transmission measurements (see Supplementary Figure 2a,c). By contrast, the dielectric tensor that we calculated from the first principles agrees significantly better. It reproduces the transmittance map on the qualitative level (see Supplementary Figure 2b) and gives an almost perfect match for the principal axis rotation angles, especially near 750 nm where the wandering principal axis phenomenon is the most pronounced. At the same time, we note some quantitative mismatch between the measured and calculated transmittance maps, which can be attributed to a slight inaccuracy of the first principle calculations for determination of the dielectric tensor.

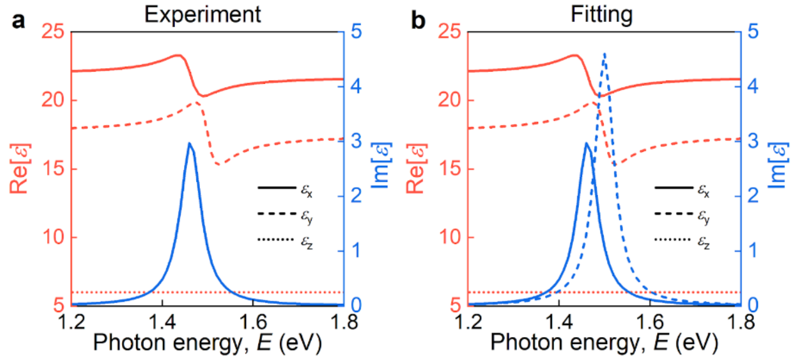

Supplementary Figure 1: Optical constants of ReS_2 from near-field measurements. a, Optical constants of ReS_2 from paper[1] and **b,** Lorentz oscillator fitting of optical constants, presented in panel (a).

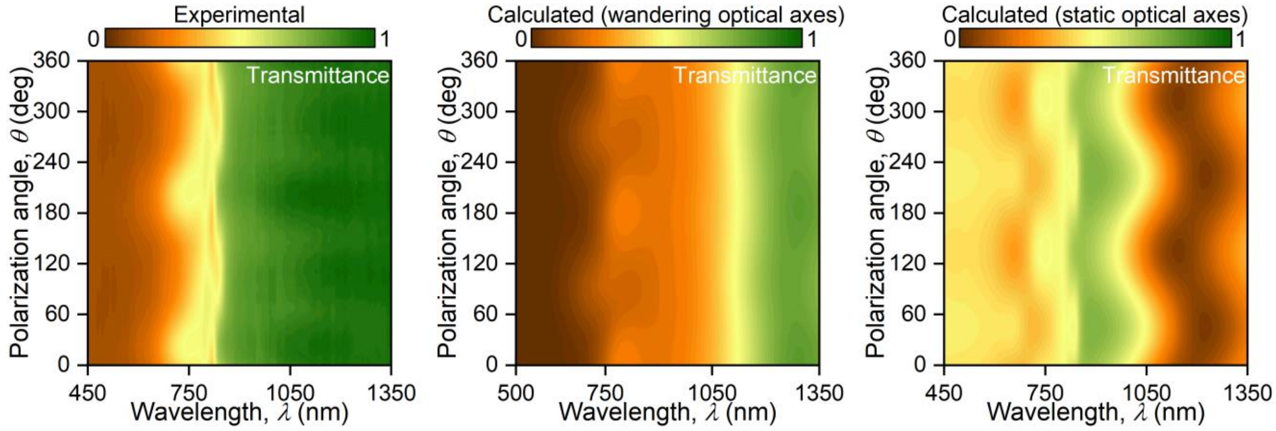

Supplementary Figure 2: Comparison of microtransmittance of ReS_2. a, Experimental transmittance for 150 nm-thick ReS_2 flake. **b**, Calculated by the transfer matrix method transmittance based on optical constants of ReS_2 from Figure 4. **c,** Calculated by the transfer matrix method transmittance based on optical constants of ReS_2 found from ellipsometry in the recent work[2].

Supplementary Note 2: Wavelength-dispersive principal optical axes in $ReSe_2$.

Supplementary Figure 3 shows the investigated $ReSe_2$ flake and corresponding polarized transmittance map. Similarly to ReS_2, $ReSe_2$ demonstrates wandering principal optical axes with variations around 55°.

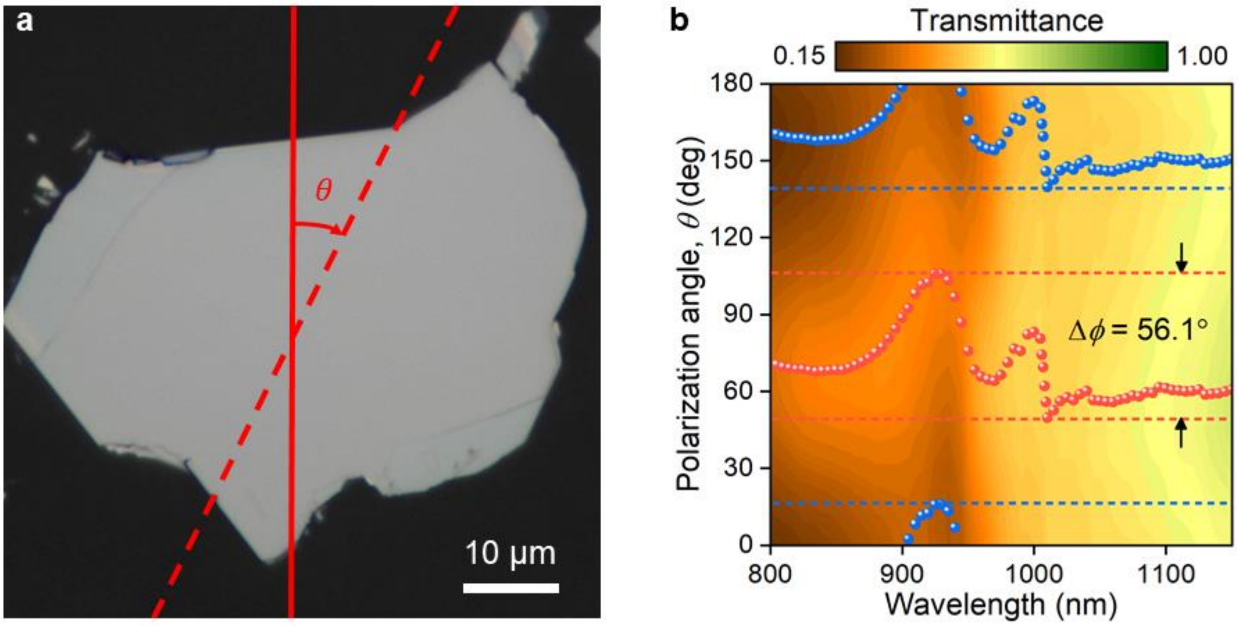

Supplementary Figure 3: **Observation of wavelength-dispersive principal optical axes in triclinic $ReSe_2$. a,** Optical image of exfoliated bulk $ReSe_2$. **b,** Polarized transmittance map. The red and blue points show the position of the principal optical axes.

Supplementary Note 3: Raman spectra for ReS_2.

To investigate the influence of principal optical axes rotation on Raman spectra of phonon modes, we measured polarized Raman at several excitation wavelength: 532 nm (Supplementary Figure 4a), 633 nm (Supplementary Figure 4b), and 780 nm (Supplementary Figure 4c). The resulting polar plots are collected in Supplementary Figures 4d-f. The comparison between the wavelength-dispersive change of both maximum intensity of phonon modes and principal optical axes rotation reveals a similar trend, as seen in Supplementary Figure 4g. Briefly, principal optical axes determine the light-matter interaction efficiency for a given polarization direction, which influences the Raman signal's intensity and results in the shift of the Raman mode's maximum intensity. The same explanation is also valid for previous Raman results, for example, in[3], the authors report a similar Raman wavelength-dependent response of polarized Raman modes of maximum intensity with respect to the *b*-axis.

Though our description is qualitative, but it already gives an insight into the wandering principal optical axes influence on the Raman signal and explains previous intriguing results[3]. Notably, the detailed Raman analysis should involve, at least, three nontrivial effects, such as wandering principal optical axes, electronic resonance effect[4], and dispersive dielectric constants[5]. Our qualitative results are summarized in Supplementary Figure 4g.

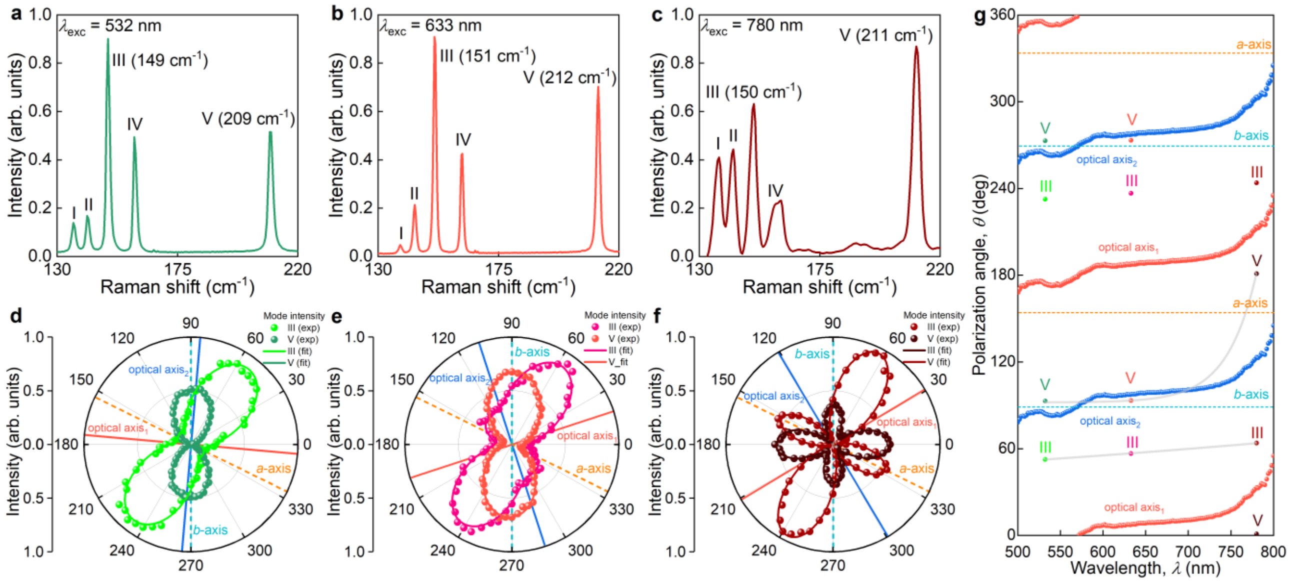

Supplementary Figure 4: Polarized Raman measurements for ReS_2. Characteristic Raman spectra for **a,** 532 nm, **b,** 633 nm, and **c,** 780 nm. Polarized phonon mode intensities of III and V phonon modes for **d,** 532 nm, **e,** 633 nm, and **f,** 780 nm. **g,** The comparison of principal optical axes rotation with phonon mode position of maximum intensity.

Supplementary Note 4: Static principal optical axes in As_2S_3.

For example of static principal optical axes, we measured the polarized transmittance map for As_2S_3 flake and determined the position of its principal optical axes (Supplementary Figure 5). We found that the maximum difference between experimental principal optical axes of As_2S_3 is only 4°, which attributes to experimental nonidealities, such as a discrete polarization angle step of 5°. Thus, As_2S_3 has static principal optical axes, unlike ReS_2 and $ReSe_2$. Therefore, we unambiguously confirm that the observed wandering principal optical axes in ReS_2 and $ReSe_2$ are uncommon for anisotropic materials.

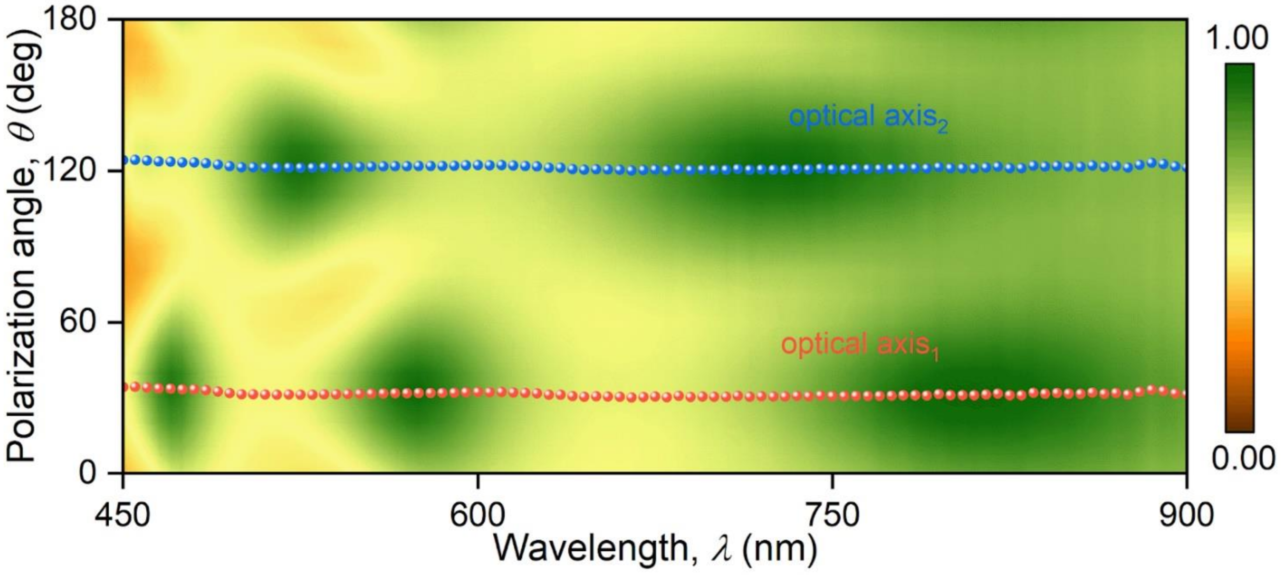

Supplementary Figure 5: Observation of static principal optical axes in As_2S_3. Polarized transmittance map for As_2S_3. The red and blue points show the position of the principal optical axes.

Supplementary Note 5: Phenomenological non-orthogonal excitons model for wandering principal optical axes in ReS_2 and $ReSe_2$.

To reproduce the observed dynamics of the principal optical axes of ReS_2, we assume that the optical response of the crystal can be attributed to the resonant excitation of two non-collinear excitonic transitions of the material. Each excitonic transition of the material contributes a Lorentzian term χ_i ($i = 1,2$) to the electric susceptibility:

$$\overleftrightarrow{\chi}_i = f_i \frac{\omega_i^2}{\omega_i^2-\omega^2-i\omega\gamma_i} \widehat{\boldsymbol{n}}_i \otimes \widehat{\boldsymbol{n}}_i^*, \tag{1}$$

where $\omega_{1,2}$ is the resonant frequency of the exciton resonance, $\gamma_{1,2}$ is its non-radiative decay rate, $f_{1,2}$ is re-scaled oscillator strength, and $n_{1,2} = \left(n_x, n_y, 0\right)^T$ is the unit vector describing the in-plane polarization of the given excitonic transition. Casimir-Onsager relations, $\varepsilon = \varepsilon^T$, guarantee that these polarization vectors are real (up to a global complex factor $e^{i\phi}$) in the absence of a static magnetic field, thus, describing linearly-polarized transitions. Therefore, we may write $n_i = (\cos\theta_i, \sin\theta_i, 0)^T$.

ReS_2 supports three exciton transitions close to the frequency range of interest around 800 nm. However, one of these transitions is rather weak; thus, we assume that only the other two excitonic transitions contribute to the observed effect. The two transitions are linearly polarized making an angle of approximately 65°. We fix the *x*-axis along the first exciton, then $n_1 = (1,0,0)^T$ and $n_2 = (\cos 65°,\ \sin 65°, 0)^T$. Adding the contributions from two independent excitonic transitions, we obtain the following permittivity tensor:

$$\overleftrightarrow{\varepsilon}(\omega) = \overleftrightarrow{\mathbb{I}}\varepsilon_\infty + \overleftrightarrow{\chi}_1 + \overleftrightarrow{\chi}_2, \tag{2}$$

where ε_∞ is the high-frequency background permittivity. The resulting parameters for the exciton model of ReS_2: $\omega_1 = 1/0.825\ \mu\mathrm{m}^{-1}$, $\omega_2 = 1/0.801\ \mu\mathrm{m}^{-1}$, $\gamma_1 = 0.05\ \mu\mathrm{m}^{-1}$, $\gamma_2 = 0.005\ \mu\mathrm{m}^{-1}$, $f_1 = 1$, $f_2 = 0.52$.

Similarly, we describe the $ReSe_2$ optical response via a three-exciton model (Supplementary Figure 6):

$$\overleftrightarrow{\varepsilon}(\omega) = \left(\varepsilon_{1\infty} + f_1 \frac{\omega_1^2}{\omega_1^2-\omega^2-i\omega\gamma_1} + f_2 \frac{\omega_2^2}{\omega_2^2-\omega^2-i\omega\gamma_2}\right) \widehat{\boldsymbol{n}}_1 \otimes \widehat{\boldsymbol{n}}_1^* + \varepsilon_{2\infty} \widehat{\boldsymbol{n}}_2 \otimes \widehat{\boldsymbol{n}}_2^* \tag{3}$$

The resulting parameters for $ReSe_2$ exciton model: $\omega_1 = 1/0.945\ \mu\mathrm{m}^{-1}$, $\omega_2 = 1/1.022\ \mu\mathrm{m}^{-1}$, $\gamma_1 = 0.045\ \mu\mathrm{m}^{-1}$, $\gamma_2 = 0.05\ \mu\mathrm{m}^{-1}$, $f_1 = 1.4$, $f_2 = 3.3$, $\varepsilon_{1\infty} = 30$, $\varepsilon_{2\infty} = 15$.

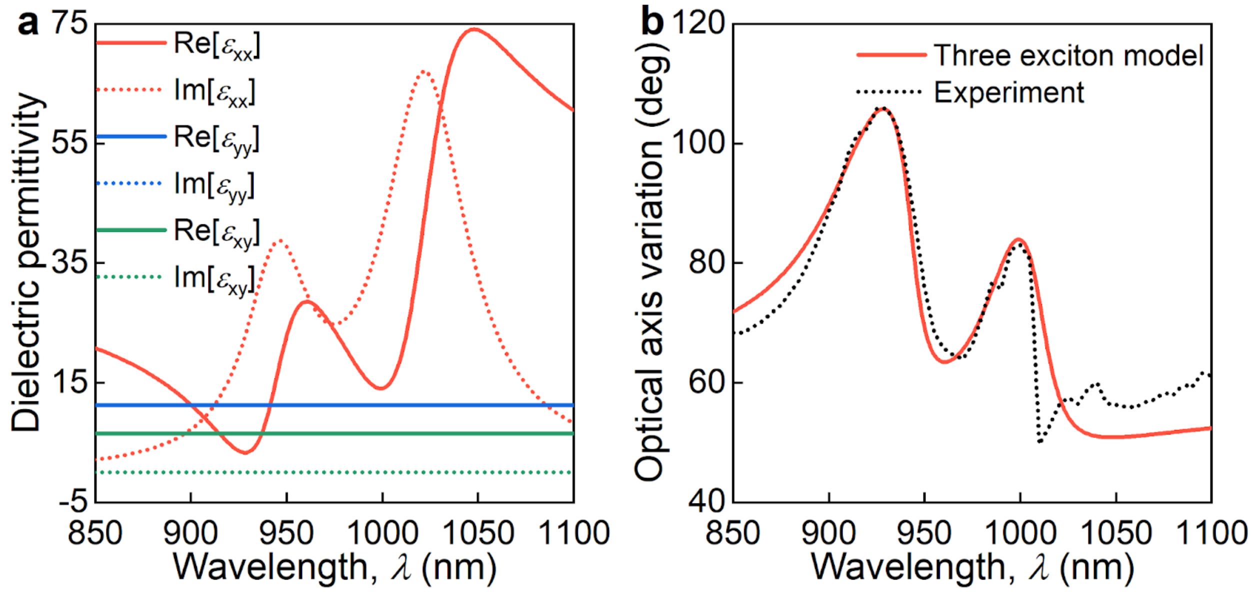

Supplementary Figure 6: Phenomenological exciton model for $ReSe_2$. a, Dielectric tensor used for the three-exciton model. **b,** Principal optical axes dependence on wavelength.

Additionally, both the Hermitian and anti-Hermitian parts of this permittivity tensor admit diagonalization in two independent orthogonal bases:

$$\frac{\overleftrightarrow{\varepsilon}+\overleftrightarrow{\varepsilon}^{\dagger}}{2} = u_1 \boldsymbol{a}_1 \otimes \boldsymbol{a}_1 + u_2 \boldsymbol{a}_2 \otimes \boldsymbol{a}_2, \quad (4)$$

$$\frac{\overleftrightarrow{\varepsilon}-\overleftrightarrow{\varepsilon}^{\dagger}}{2} = v_1 \boldsymbol{b}_1 \otimes \boldsymbol{b}_1 + v_2 \boldsymbol{b}_2 \otimes \boldsymbol{b}_2, \quad (5)$$

where a_i и b_i are real. Due to the Casimir-Onsager relation, the Hermitian part of the tensor is the symmetric matrix with real elements, whereas the anti-Hermitian is the symmetric matrix with imaginary elements. According to **Figure 4** of the main text, the imaginary parts of the dielectric permittivity tensor components are much lower than the real parts in the range of consideration. Consequently, we assume that the principal optical axes of the Hermitian part approximately coincide with the principal optical axes extracted from the transmittance measurements.

Supplementary Note 6: Angular dependance of weak confinement modes in the reflection scheme of near-field microscopy.

Supplementary Figure 7 shows near-field measurements of the $ReSe_2$ disk with a thickness of 125 nm in reflection mode. The strong angular dependence, which can be seen in Supplementary Figure 7 is caused by the geometry of the experiment. This effect is related to momentum conservation along the edge direction[1,6]. This effect is associated with a different phase shift of the light scattered by the tip of the probe and the distant object (edge of the disk). Light focused on the tip of the probe is converted into evanescent waves, which, upon reaching the sample-air interface, are divided into two beams: one part is elastically reflected from the surface then scattered again on the probe and returned to the detector, the other part excites cylindrical waveguide modes in the sample, which scatter upon reaching the edge of the sample (see

Supplementary Figure 8a). Since a parabolic mirror collects the light at an angle relative to the normal of the sample, an additional phase shift occurs between these two parts of the light due to different propagation lengths in the air. The phase shift depends on the distance *x* from the edge of the sample to the tip, thus, the interference between those two beams changes the observed oscillation frequency of the waveguide mode. The effect can be expressed as:

$$n_{eff} = n_{SNOM} + \cos(\gamma)\sin(\beta)$$

where γ is the angle between the illumination wavevector k and its projection $k_{\parallel}$ on the sample surface plane, and β is the angle between $k_{\parallel}$ and the sample edge. Thus, the sinusoidal dependence shown in Supplementary Figure 7 is due to the change of the position of the sample edge on which scattering occurs. It is important to note that in this case, both parts of the scattered light (from the probe and from the edge) behave as near-field: they quickly decay as the probe moves away from the surface. In the case of transmittance measurements (see Methods in the main text), we illuminate the sample along the normal from below focusing light on the hole. Using synchronized movement of the sample and the lower parabolic mirror relative to the probe and the upper parabolic mirror[7], we can ensure that the light always remains focused at the hole, and does not move with the probe, as in the case of reflection. When light is scattered by a hole, one part of the scattered radiation goes directly to the detector, the other part couples into waveguide modes, and after passing through the sample, is scattered by the probe, as seen in Supplementary Figure 8b. It is important to note that although such a system is very similar to the system described above, the interference effect responsible for the spatial frequency shift will not occur since the radiation directly scattered by the hole to the detector does not behave like near-field radiation and does not diminish when the tip of the probe moves away from the sample. Therefore, it will be filtered out by a heterodyne system[8] and will not be observed in the background-free images (harmonics ≥ 3). However, in images that are not completely free from background (harmonic = 1), the radiation that comes directly from the hole is not suppressed completely, and it is possible to observe frequency shift effect similar to that in the reflection mode (see Supplementary Figure 8c-d). It is important to point out that even in the image with significant background it is still possible to observe angular dependence related to the anisotropy of the material (~sin(2α)), however according to our calculations this effect is more than 2.5 times weaker than spatial frequency shift related to the interference with background (~sin(α)). Accordingly, in the background-free transmittance images, we do not observe angular dependance of ~sin(α), and instead, we observe ~sin(2α) dependence, which comes from the in-plane anisotropy of the material. The absence of the spatial frequency shift in the background-free transmittance measurements is one of the main reasons why we carried out our main measurements using this technique.

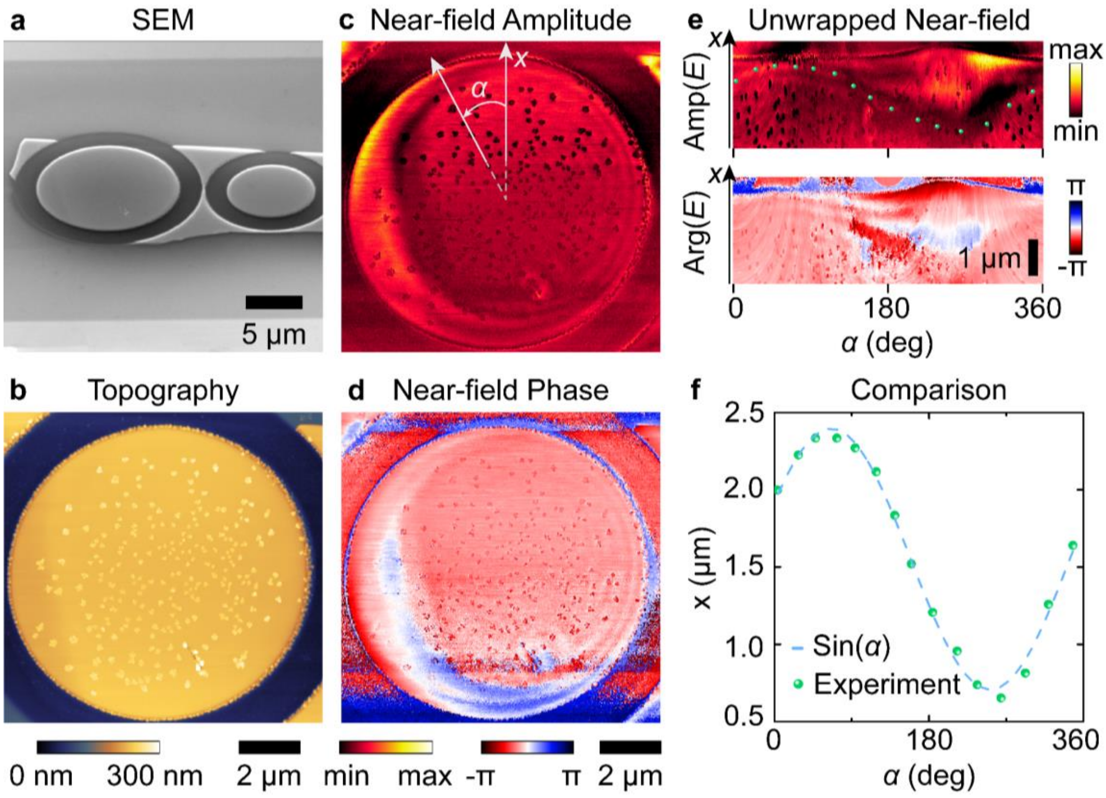

Supplementary Figure 7: Near-field microscopy of $ReSe_2$ disk in reflection mode. **a**, Scanning electron microscopy image of the sample. **b**, atomic force microscopy image of the measured $ReSe_2$ disk. **c-d**, amplitude and phase of the near-field signal measured in the reflection mode. **e**, unwrapped, relative to the center, measurements from **c** and **d**, the green points in panel e indicate profile dependance of the mode on the in-plane angle of rotation. **f**, comparison of the mode profile picked from panel **e** and sinusoidal dependence on the angle α.

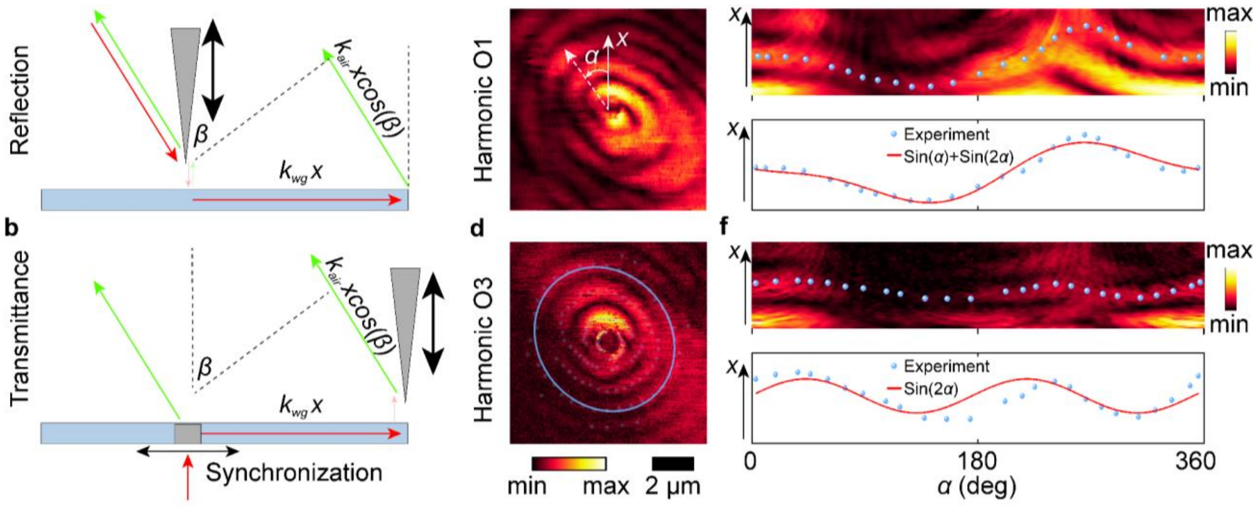

Supplementary Figure 8: Explanation of difference between transmittance and reflection modes in near-field microscope. **a-b**, Reflection and transmittance schemes in near-field microscopy. **c-d**, near-field image with first and third harmonics filtration. **e-f**, unwrapped, relative to the center, measurements from **c** and **d**, the blue points indicate the dependance of the mode profile on the in-plane angle of rotation, the red curve represents a sinusoidal fir with arguments α and 2α, respectively.

Supplementary Note 7: Theoretical background of waveguide mode direction change – computational approach.

In order to describe near-field distribution in the vicinity of the circular hole in the $ReSe_2$ layer, we numerically solve the appropriate scattering problem in COMSOL Multiphysics. In our case, the scattering element (hole) is embedded not in a homogeneous environment but in a complex layered structure, which prevents us from applying standard, built-in computational techniques.

Nevertheless, the widely spread scattered field formulation technique[9,10] allows us to solve the considered scattering problem in two simple steps briefly summarized below:

1) In the first stage, we consider only the background (air/$ReSe_2$/glass) layered structure. Then, we solve the Maxwell equations for a normally incident light of arbitrary polarization incident from the glass substrate. In principle, this subproblem might be solved fully analytically, but for convenience and potential scalability, we do it numerically in COMSOL Multiphysics. The obtained background electromagnetic fields (magnetic $\boldsymbol{H}_{\rm bg}$ and electric $\boldsymbol{E}_{\rm bg}$ fields) solve the equation:

$$\nabla \times \boldsymbol{H}_{\rm bg} = -ik_0\hat{\varepsilon}_{\rm bg}\boldsymbol{E}_{\rm bg}, \tag{6}$$

where k_0 is the vacuum wavevector and $\hat{\varepsilon}_{\rm bg} = \hat{\varepsilon}_{\rm bg}(z)$ is a permittivity tensor, which is a piecewise-constant function changing from layer to layer.

2) Next, we consider the same field incident on a structure with a hole in the $ReSe_2$ layer. Total electromagnetic fields (magnetic $\boldsymbol{H}_{\rm tot}$ and electric $\boldsymbol{E}_{\rm tot}$ fields) solve the similar equation:

$$\nabla \times \boldsymbol{H}_{\rm tot} = -ik_0\hat{\varepsilon}\boldsymbol{E}_{\rm tot}, \tag{7}$$

where permittivity $\hat{\varepsilon} = \hat{\varepsilon}(\vec{r})$ is a function of radius-vector and describes both the layer structure and the hole itself. In turn, we can represent the total field as a sum of the background (magnetic $\boldsymbol{H}_{\rm bg}$ and electric $\boldsymbol{E}_{\rm bg}$ fields) and scattered (magnetic $\boldsymbol{H}_{\rm sc}$ and electric $\boldsymbol{E}_{\rm sc}$ fields) fractions:

$$\boldsymbol{H}_{\rm tot} = \boldsymbol{H}_{\rm bg} + \boldsymbol{H}_{\rm sc}, \tag{8}$$

$$\boldsymbol{E}_{\rm tot} = \boldsymbol{E}_{\rm bg} + \boldsymbol{E}_{\rm sc}, \tag{9}$$

$$\nabla \times (\boldsymbol{H}_{\rm bg} + \boldsymbol{H}_{\rm sc}) = -ik_0\hat{\varepsilon}(\boldsymbol{E}_{\rm bg} + \boldsymbol{E}_{\rm sc}), \tag{10}$$

Substituting Eq. 6 in Eq. 10 provides us with expressions suitable for calculating the scattered field:

$$\nabla \times \boldsymbol{H}_{\rm sc} = \frac{4\pi}{c}\boldsymbol{j} - ik_0\hat{\varepsilon}\boldsymbol{E}_{\rm sc}, \tag{11}$$

$$\frac{4\pi}{c}\boldsymbol{j} = -ik_0(\hat{\varepsilon} - \hat{\varepsilon}_{\rm bg})\boldsymbol{E}_{\rm bg}, \tag{12}$$

These expressions show that the scattered field might be found as a result of the radiation of the current $\boldsymbol{j}$, which is proportional to the background electric field $\boldsymbol{E}_{\rm bg}$ and the difference permittivities between the total and background structure $\Delta\hat{\varepsilon}(\boldsymbol{r}) = \hat{\varepsilon}(\boldsymbol{r}) - \hat{\varepsilon}_{\rm bg}(\boldsymbol{r})$, which is non-zero only inside the volume of the hole. Knowing the background field distribution from the first step, we numerically find the scattered field by solving the radiation problem (see Eqs. 11-12) in COMSOL Multiphysics. The total electric field is finally found by adding the background field to the scattered one.

Finally, it is worth noting that the described numerical approach does not account for the influence of the near-field microscope tip. Nevertheless, the near-field distributions calculated appropriately characterize the studied samples and capture the main peculiarities as it is shown in subsequent Notes.

Supplementary Note 8: Theoretical background of waveguide mode's direction change – near-field calculations analysis.

We consider a 150-nm-thick $ReSe_2$ layer on a glass substrate in our numerical near-field calculations. The refractive index of the glass is 1.4, whereas the permittivity of $ReSe_2$ is described by the phenomenological 3-exciton model (see Supplementary Note 5 and Supplementary Figure 6 for details). We illuminate a 500-nm-radius hole in the layer by a plane wave normally incident from the substrate and study the z-component (perpendicular to the structure surface) of the electric field, $|E_z|$, in the air at a distance of 5 nm from the air/$ReSe_2$ interface. Due to the peculiar structure of the 3-exciton model's permittivity tensor $\left(\hat{\varepsilon}_{xz} = \hat{\varepsilon}_{yz} = 0\right)$, the normally incident plane wave does not induce a z-component in the background field, $E_{bg}^{z} = 0$. Therefore, the z component of the total electric field equals to the corresponding component of the scattered field, $E_{tot}^{z} = E_{sc}^{z}$.

Supplementary Figure 9 presents a series of near-field distributions, categorized by the incident light wavelengths (columns) and angles of the flake's rotation (rows). The three columns represent wavelengths of 920 nm, 940 nm, and 950 nm, while the seven rows display fields for rotation angles of {0°, 30°, 60°, 90°, 120°, 150°, 180°}. Both 0° and 180° rotations yield identical results and are shown for the ease of comparison with adjacent panels. The incident light polarization (indicated by white arrows) is vertical in all panels.

Upon comparing the columns, we observe that the slight rotation of the bright (yellow in the image) electrostatic-dipole-like-polarized field in the immediate vicinity of the hole is most apparent in the first and last rows. The waves surrounding the hole also exhibit slight shifts. Concurrently, the flake's rotation has a relatively weak effect on the bright, dipole-like field, which remains predominantly vertically polarized regardless of the flake's orientation. However, the waves surrounding the hole clearly rotate along with the flake. This supports the notion that the observed waves are associated with the waveguide modes of the anisotropic $ReSe_2$ layer.

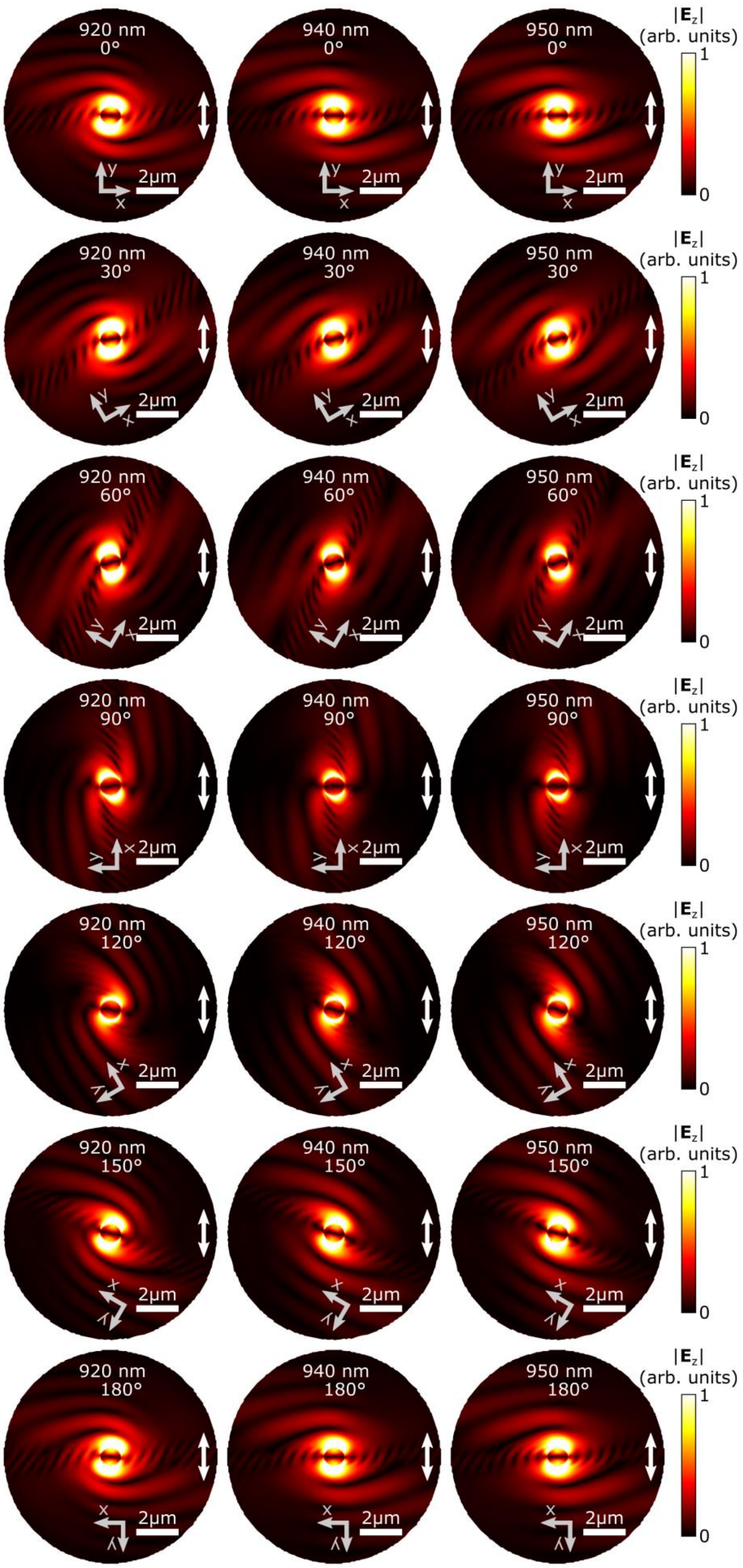

920 nm
940 nm
950 nm
0°
30°
60°
90°
120°
150°
180°
|$\mathbf{E}_z$|
(arb. units)
1
0
2μm
x
y

Supplementary Figure 9: Near-field distribution near a circular hole in the $ReSe_2$ layer. Three columns correspond to 920 nm, 940 nm, and 950 nm wavelengths. Eight rows correspond to six linear polarizations of incident light taken with a 30-degree step, left-handed (LCP), and right-handed (RCP) circular polarization. Near field distribution pattern in the considered anisotropic structure mainly depends on the polarization of the incident beam, but also, slightly varies with wavelength.

The numerical calculations not only provide the reader with the absolute value of the electric field but also, its phase (see Supplementary Figure 10). To analyze them together, we employ a straightforward data processing algorithm, illustrated for the case of a non-rotated flake and a wavelength of 920 nm in Supplementary Figure 10. The algorithm consists of the following steps: *(i)* transformation of coordinates from (x, y) to (r, φ) (see Supplementary Figure 10); *(ii)* referencing to[1,11], and application of the Parzen window (see the window shape in Supplementary Figure 10) to smoothen the computational domain's edge and the hotspot near the hole (see Supplementary Figure 10); and finally, *(iii)* performing a Fourier transform on the obtained complex field for the radial coordinate and analyzing its absolute value (see Supplementary Figure 10). The application of the Parzen window enables us to extend the Fourier integral to infinite limits and avoid parasitic oscillations caused by the Gibbs phenomenon.

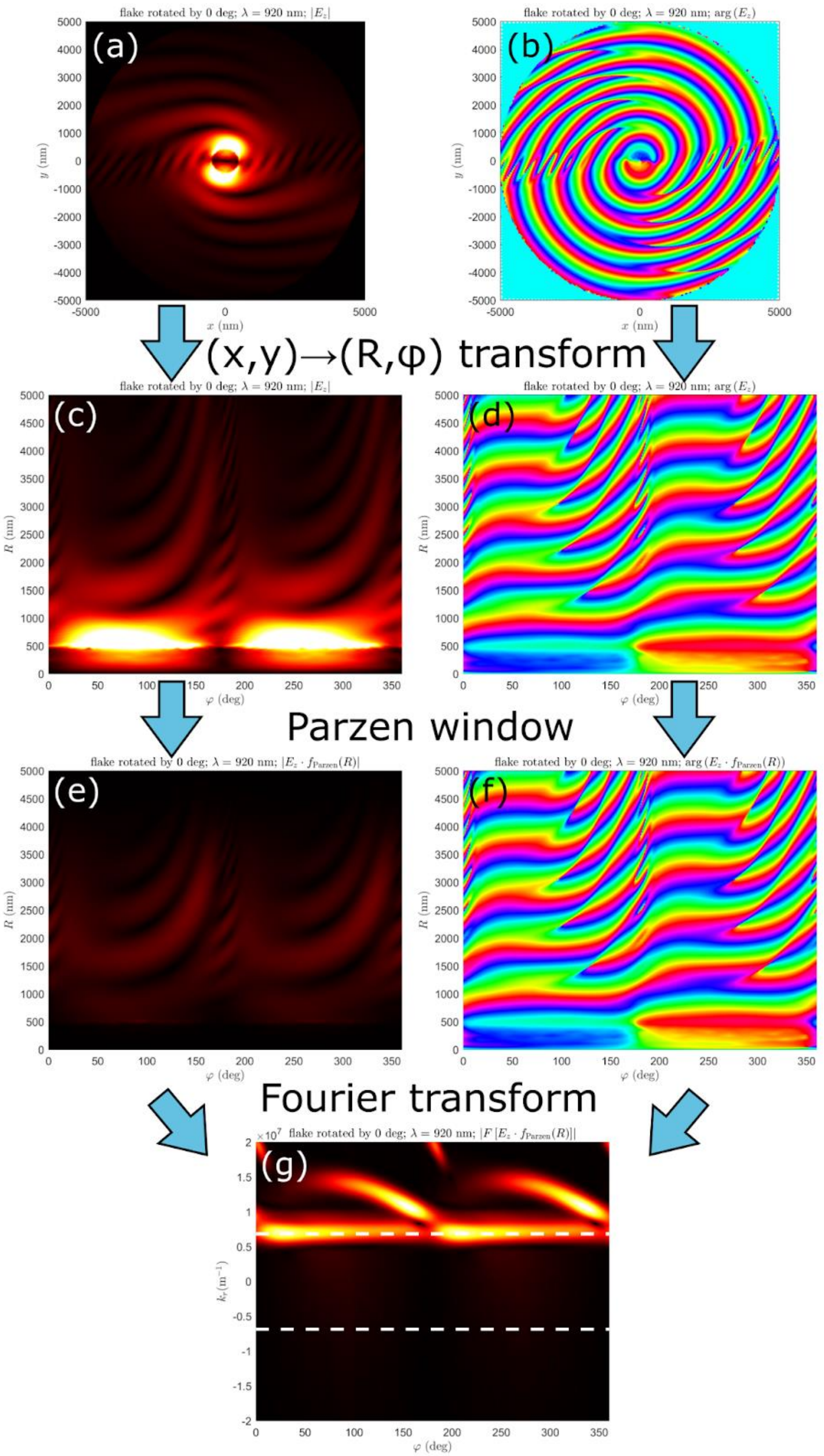
flake rotated by 0 deg; λ = 920 nm; |E_z|
(a)
flake rotated by 0 deg; λ = 920 nm; arg(E_z)
(b)
y (nm)
x (nm)
(x,y)→(R,φ) transform
(c)
(d)
R (nm)
φ (deg)
Parzen window
flake rotated by 0 deg; λ = 920 nm; |E_z · f_Parzen(R)|
(e)
flake rotated by 0 deg; λ = 920 nm; arg(E_z · f_Parzen(R))
(f)
Fourier transform
flake rotated by 0 deg; λ = 920 nm; |F[E_z · f_Parzen(R)]|
(g)
×10^7
k_r (m^-1)

Supplementary Figure 10: Processing of the near-field distribution for a circular hole in the $ReSe_2$ layer. a, The absolute value and **b,** phase of the near-field distribution are shown for vertical polarization of 920 nm light incident on a non-rotated flake. **c-d,** The same fields after transformation to polar coordinates. **e-f,** Multiplication of the obtained field with a real-valued Parzen window does not affect the phase, but smooths the absolute value of the field near the hole and edges of the computational domain. **g,** The absolute value of the obtained field Fourier image. The white dashed line indicates the wavevector of air modes, and the observed bright lines indicate the dispersion of air and waveguide modes.

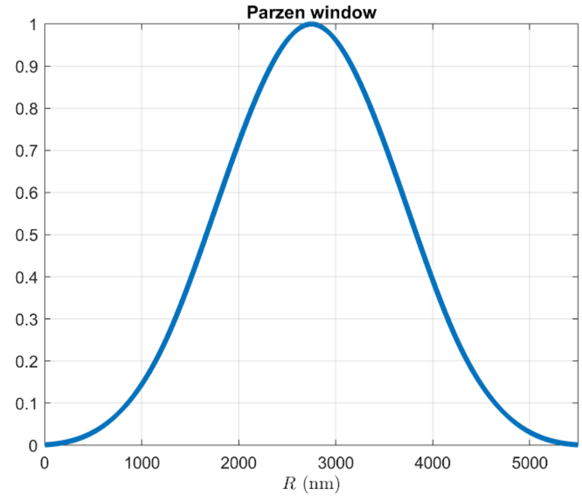

Supplementary Figure 11: The shape of the Parzen filter applied to the near-field distributions.

The procedure described above allows us to effectively distinguish the contributions of different modes to the overall field. As depicted in Supplementary Figure 10, all the modes are situated in the upper half-space, indicating positive values of the radial wavevector, k_r. This confirms the evident fact that the modes propagate outward from the hole. One of the branches follows the horizontal white dashed line, which corresponds to the light wavevector in the air. This confirms that this particular mode is associated with freely propagating air modes scattered out by the hole. Moreover, the angle dependence of this mode's amplitude exhibits a typical vertical dipole radiation pattern – with dips at $\varphi_{deeps} = \{0°, 180°\}$ and peaks in between. However, the peaks are significantly shifted from the expected positions of ±90°, as the actual polarization currents induced by the hole differ significantly from a point dipole that is vertically aligned with the polarization of the incident light. In addition to the air modes, we also observe branches of waveguide modes of two types. The first type exhibits strong angular dispersion and propagates in almost all possible directions, while the second type is observed in a narrow range of angles near $\varphi = \{0°, 180°\}$ and possesses much larger wavevectors.

Based on our findings regarding the mode structure, we conclude that the waves observed in Supplementary Figure 9 (as well as in Supplementary Figure 10) are not associated with either air modes or waveguide modes individually, but rather with their interference: $|E_z(\varphi, r)| \propto \left|E_{air}(\varphi)e^{ik_0 r} + E_{waveguide}(\varphi)e^{ik_{wg} r}\right|$. Therefore, the radial period of these waves is inversely proportional to the difference between their wavevectors, given by $\lambda_{waves} = \frac{2\pi}{k_{wg}-k_0} = \frac{\lambda_{wg}\lambda_0}{\lambda_0-\lambda_{wg}} = \frac{\lambda_0}{\beta_{wg}-1}$, where β_{wg} represents the index of the waveguide mode and $\lambda_{waves}, \lambda_{wg}, \lambda_{wg}$ correspond to the period of the waves in the near-field, wavelengths of the waveguide, and air modes, respectively.

By following the described procedure, we obtain the Fourier spectra (refer to Supplementary Figure 12) for various wavelengths and angles of flake rotation. All the observed effects are consistent with what we have already observed in real space (see Supplementary Figure 9), but they are much more straightforward. Notably, the transformation of the angular dispersion of the waveguide modes with wavelength becomes more apparent. The rotation of the flake results in a corresponding rotation of the waveguide modes, while the angular position of the "dipole-like" air mode remains mostly unaffected.

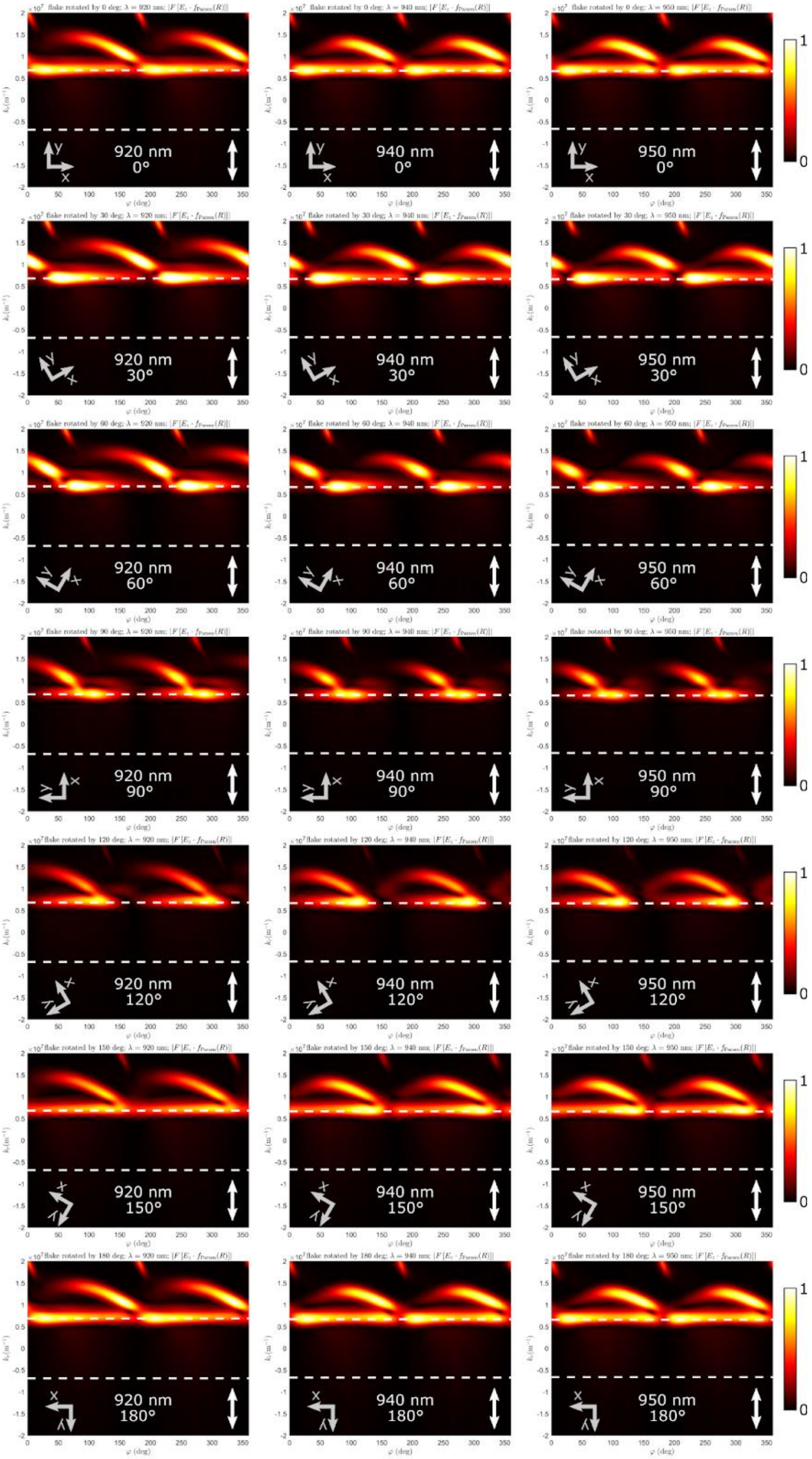

Supplementary Figure 12: Fourier spectra of the near-field distribution for a circular hole in the $ReSe_2$ layer. Each panel demonstrates the absolute value of the Fourier transform of the filtered near field, $|F[E_z(\varphi, r) \cdot f_{Parzen}(r)](\varphi, k_r)|$. The three columns correspond to wavelengths of 920, 940, and 950 nm, while the even rows correspond to flake rotations of 0°, 30°, 60°, 90°, 120°, 150°, and 180°. The gray axes indicate the orientation of the flake, and the white vertical arrows show the polarization of the incident light. The white dashed horizontal lines correspond to the wavevector of air modes $k_0 = \pm\frac{2\pi}{\lambda_0}$.

Supplementary Note 9: Comparison of calculated and measured near-fields.

In the previous Note, we discussed the contribution of air modes and waveguide modes to the near field and explained the observed waves through their interaction. To analyze this interaction, we utilized both the absolute value and phase of the electric field. However, due to limitations in experimental measurements, we are unable to obtain a reliable phase of the measured signal. As a result, direct comparison between the Fourier spectra of calculated fields (see Supplementary Figure 12) and processed experimental data is not possible. To address this issue, we applied the same postprocessing procedure to the calculated fields, considering only their absolute values and disregarding the phase (see Supplementary Figure 13). Since the absolute value of an electric field is a real-valued function, its Fourier image is an even function of the wavevector (see Supplementary Figure 13). The absolute value of the near-field does not differentiate between the contributions of air and waveguide modes. Consequently, in the spectra, we only observe the lines corresponding to the waves observed in the near field, which result from the interference of these modes. As mentioned earlier, the wavevector of these modes is determined by the difference $|k_{waves}| = k_{wg} - k_0$. In other words, waveguide modes are "shifted" by a wavevector k_0 towards the origin, while still exhibiting angular dispersion similar to the original waveguide modes. Additionally, the bright horizontal line at $k_r = 0$ is associated with the "mean background" field, which is determined by the amplitudes of both air and waveguide modes, as well as other non-resonant contributions of the scattered field.

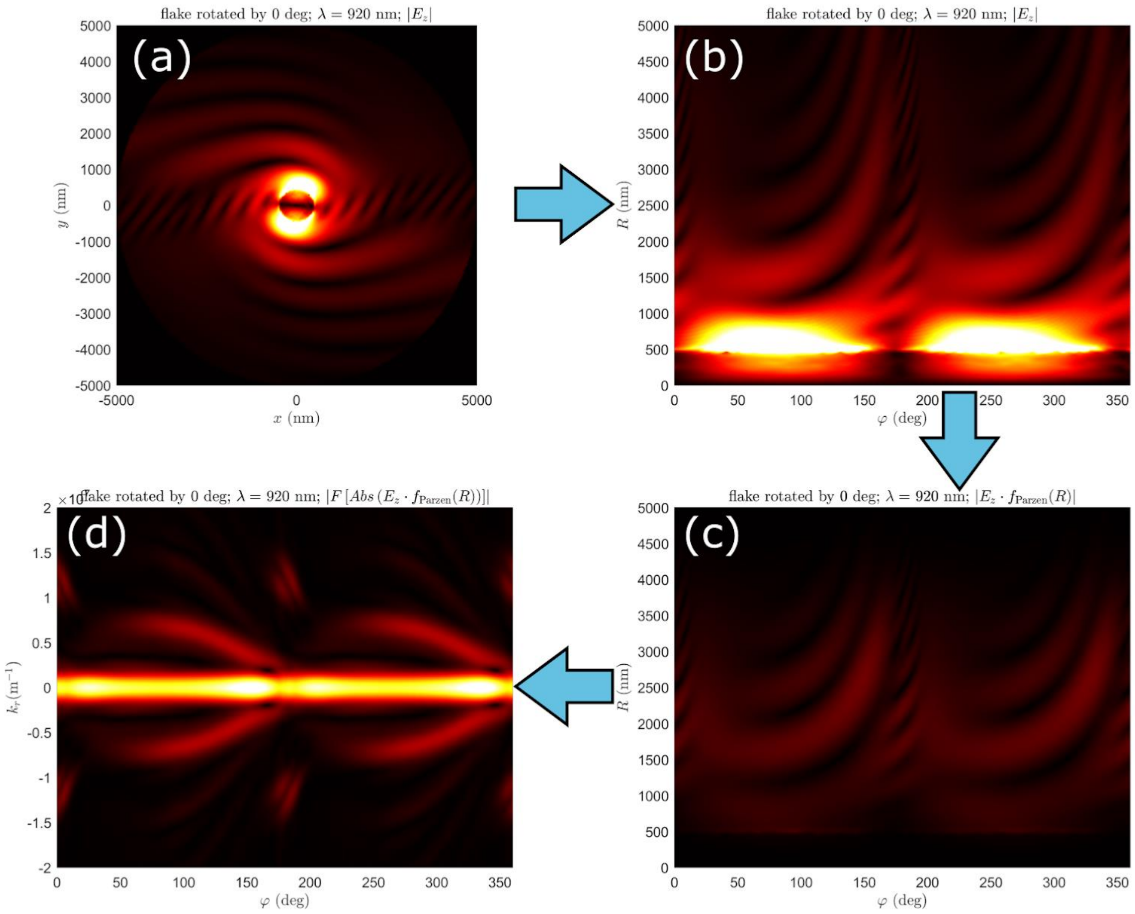

Supplementary Figure 13: Processing of the near-field distribution for a circular hole in the $ReSe_2$ layer. a, The absolute value of the near-field distribution is shown for vertical polarization of 920 nm light incident on a non-rotated flake. **b,** The same fields after transformation to polar coordinates. **c,** Multiplication of the obtained field with a real-valued Parzen window smooths the absolute value of the field near the hole and edges of the computational domain. **d,** The absolute value of the obtained field Fourier image. The observed bright lines are associated with the mean background field ($k_r = 0$) and interference of waveguide and air modes.

We repeated the procedure for different values of wavelength and flake orientations, as shown in Supplementary Figure 14. Although the Fourier spectra of the absolute value of the near field appear slightly different from the previously demonstrated ones, all the described effects are still present. The waveguide modes still rotate with the flake, while the background-associated harmonics are more stable.

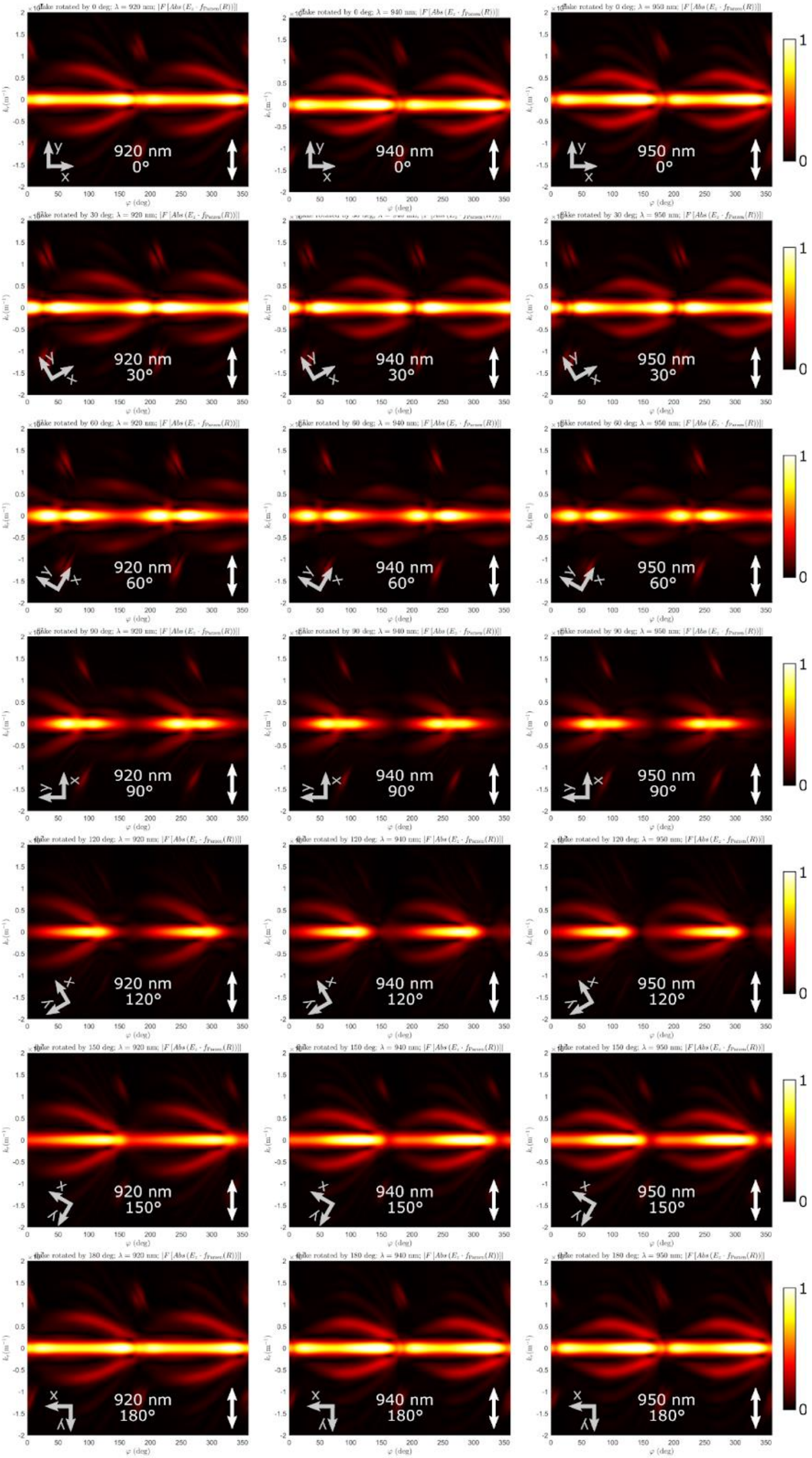
920 nm
0°
940 nm
0°
950 nm
0°
920 nm
30°
940 nm
30°
950 nm
30°
920 nm
60°
940 nm
60°
950 nm
60°
920 nm
90°
940 nm
90°
950 nm
90°
920 nm
120°
940 nm
120°
950 nm
120°
920 nm
150°
940 nm
150°
950 nm
150°
920 nm
180°
940 nm
180°
950 nm
180°
φ (deg)
1
0

Supplementary Figure 14: Fourier spectra of the near-field distribution for a circular hole in the $ReSe_2$ layer. Each panel demonstrates the absolute value of the Fourier transform of the filtered near-field absolute value, $|F[E_z(\varphi, r) \cdot f_{Parzen}(r)](\varphi, k_r)|$. The three columns correspond to wavelengths of 920, 940, and 950 nm, while the even rows correspond to flake rotations of 0°, 30°, 60°, 90°, 120°, 150°, and 180°. The gray axes indicate the orientation of the flake, and the white vertical arrows show the polarization of the incident light.

A comparison between the numerical estimations and experimental results can be found in Supplementary Figure 15. Our calculations reproduce the main experimental peculiarities, such as suppressed scattering in the first and third quadrants and a slight increase in the period of the waves with angle in the upper and lower semispaces.

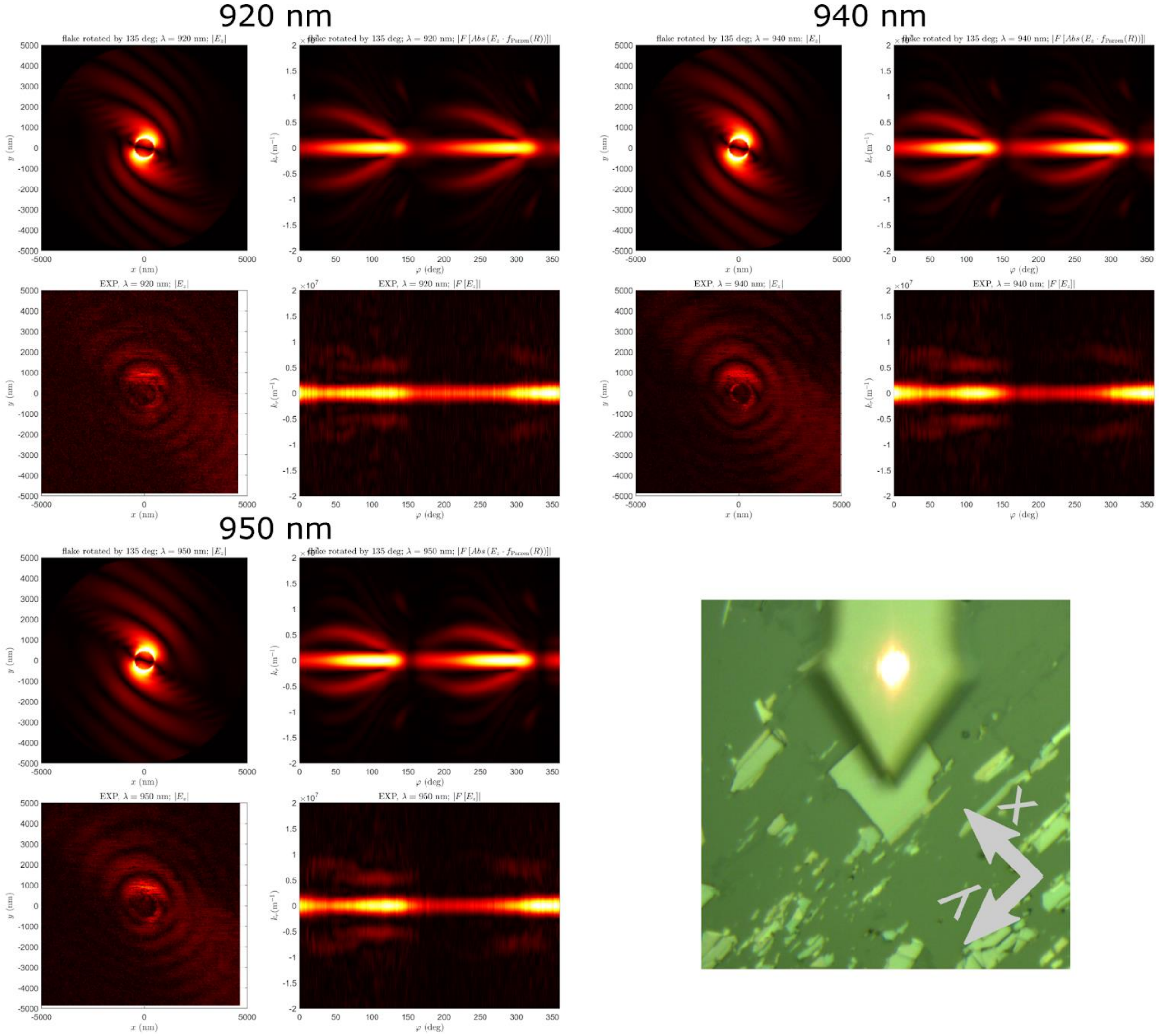

Supplementary Figure 15: Comparison of the numerically calculated and experimentally measured near-field distributions for a circular hole in the $ReSe_2$ layer. The comparison is shown for wavelengths of 920, 940, and 950 nm of incident light for both real-space distribution and corresponding Fourier spectra. A photograph demonstrates the orientation of the flake in the microscope.

Supplementary Note 10: Isotropic approximation for determination of ReS_2 optical constants.

To describe the optical response of ReS_2, we use isotropic approximation, where we assume an isotropic optical model to fit polarization spectra. In other words, for fixed polarization angle and wavelength, we determined refractive index *n* and extinction coefficient *k*, which reproduce reflectance *R*, transmittance *T*, and absorbance $A = 1 - R - T$ spectra, shown in Supplementary Figure 16, for given polarization angle and

wavelength. The resulting refractive indices and extinction coefficients are collected in Supplementary Figure 16.

In addition, we measured unpolarized transmittance and reflectance (Supplementary Figure 17c) to obtain average optical constants (Supplementary Figure 17f) from their point-by-point fitting. As a result, we find the refractive index and extinction coefficient for a given wavelength, which gives experimental unpolarized reflectance and transmittance (Supplementary Figure 17c) for this wavelength. However, the resulting optical constants do not follow Kramers-Kronig relations in contrast to other anisotropic vdW materials[2,12]. In particular, we observe only one exciton peak around 815 nm, whereas, in the refractive index, we have two inflection points in Supplementary Figure 17f. A similar trend was also observed for ReS_2 (see Supplementary Note 1)[1]. Consequently, ReS_2 and $ReSe_2$ exhibit anomalous optical responses even for unpolarized light due to wavelength-dispersive principal optical axes.

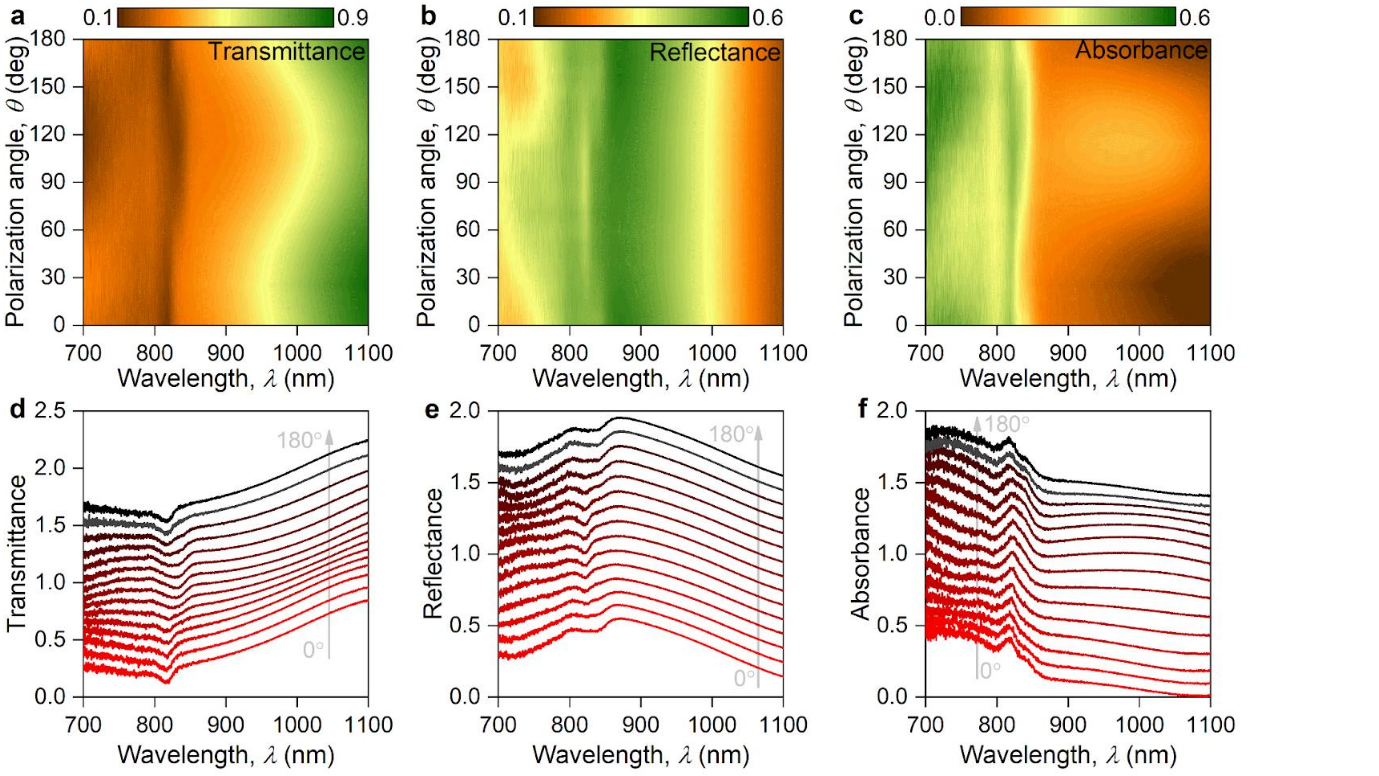

Supplementary Figure 16: Polarized spectra of ReS_2 flake. Polarized **a,** transmittance, **b,** reflectance, and **c,** absorbance map. Polarized **d,** transmittance, **e,** reflectance, and **f,** absorbance spectra. Graphs in panels (d-f) are shifted by 0.1 for clarity.

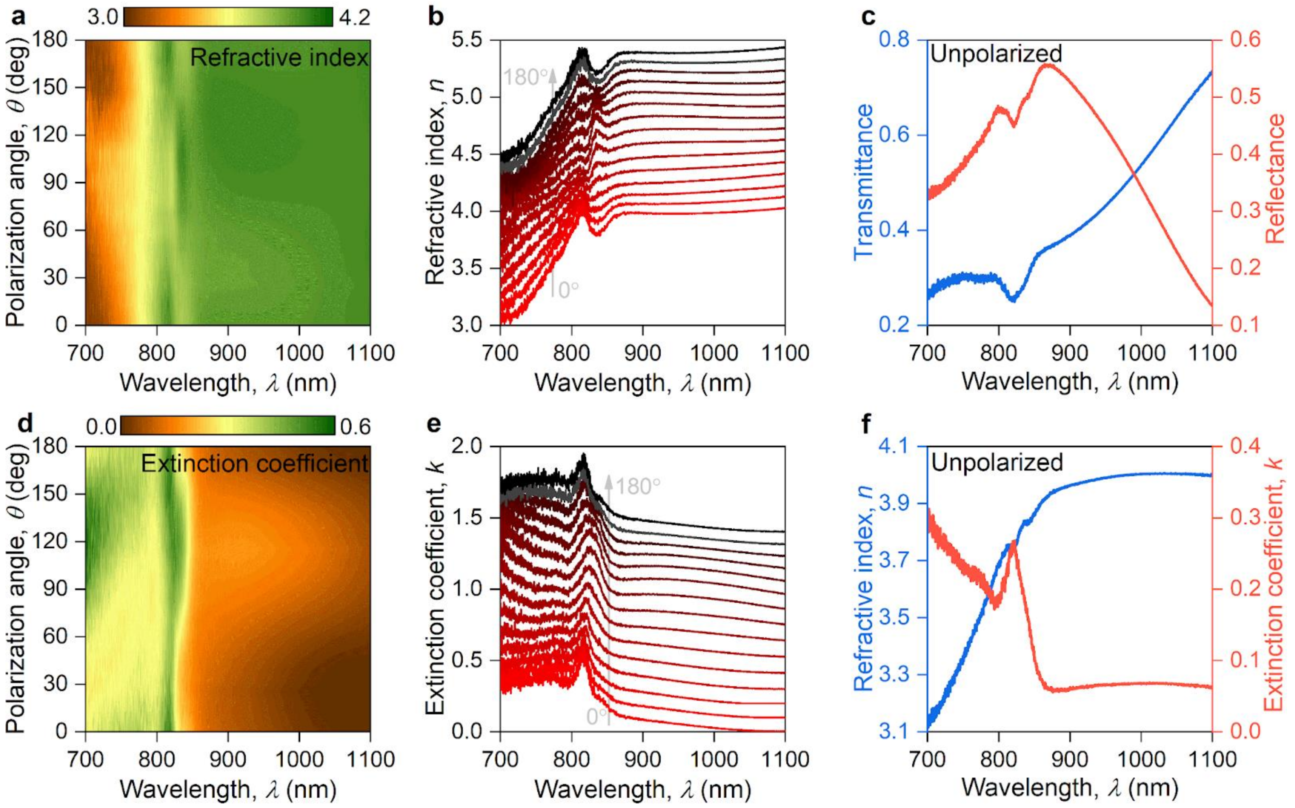

Supplementary Figure 17: Isotropic approximation for ReS_2 optical constants. a, Polarized effective refractive index map. **b,** Effective refractive index dispersion in dependence with polarization angle. Graphs are shifted by 0.1 for clarity. **c,** unpolarized transmittance and reflectance spectra. **d,** Polarized effective extinction coefficient map. **e,** Effective extinction coefficient in dependence with polarization angle. Graphs are shifted by 0.1 for clarity. **f,** Average refractive index and extinction coefficient from a point-by-point fit of unpolarized transmittance and reflectance from panel (c).

Supplementary Note 11: First-principle calculations for $ReSe_2$.

Supplementary Figure 18 shows first-principle calculations of anisotropic optical constants of $ReSe_2$. Moreover, excitonic effect and bandgap of ReS_2 may change with thickness, especially for monolayer, bilayer, and trilayer cases[13]. However, another recent work[14] demonstrates that ReS_2 has one of the smallest dependecies of optical and electronic properties on the number of atomic layers among vdW materials owing to Peierls distortion of its 1T crystal structure, which prevents ordered stacking and minimizes the interlayer overlap of wavefunctions. As a result, we expect that the observed behavior/extent of the wandering principal optical axes for monolayer, bilayer, and trilayer ReS_2 will be similar to its bulk. Additionally, we present first-principle calculations of anisotropic permittivity tensor of monolayer (Supplementary Figure 19), bilayer (Supplementary Figure 20), and trilayer (Supplementary Figure 21) ReS_2 that show similar optical response similar to bulk ReS_2 in Figure 4 of the main text.

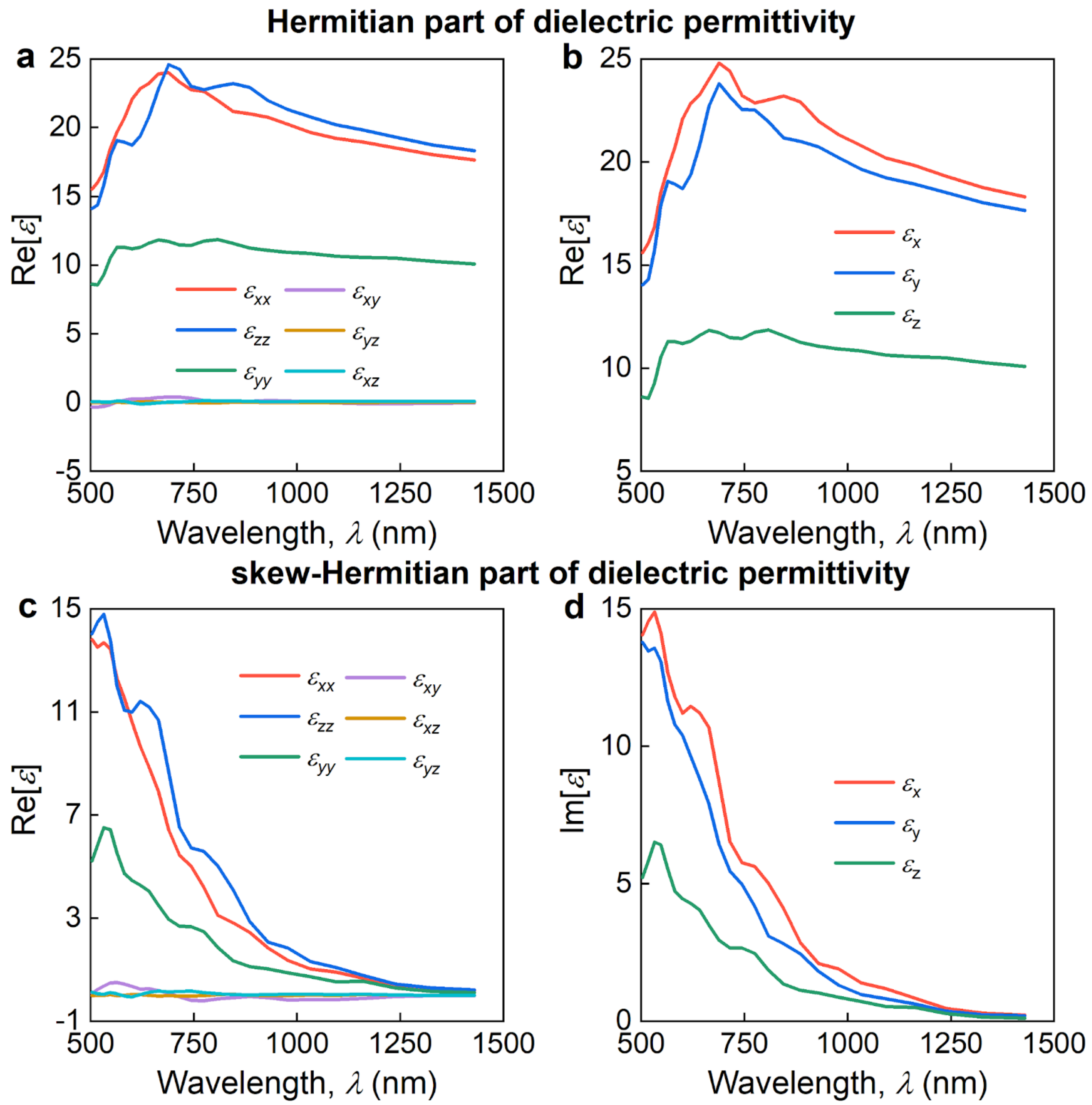

Supplementary Figure 18: First-principle calculations of $ReSe_2$ dielectric tensor. a, Hermitian and **c**, skew-Hermitian parts of dielectric tensor. **b**, Hermitian and **d**, skew-Hermitian components of dielectric permittivity after diagonalization.

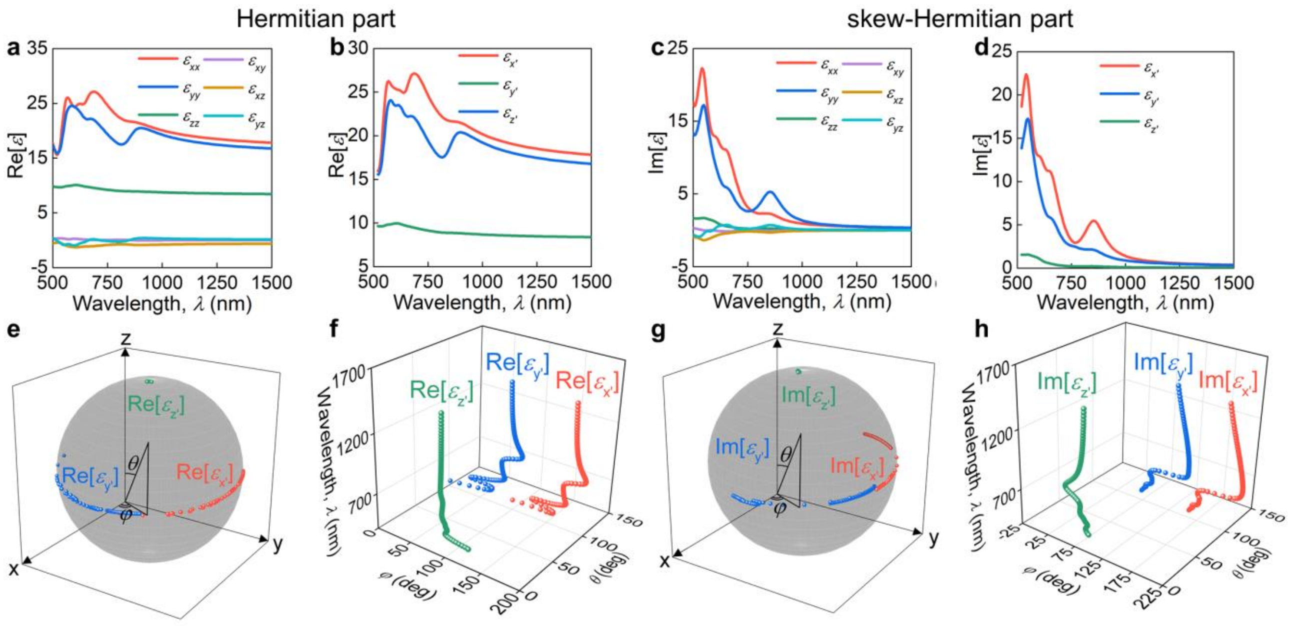

Supplementary Figure 19: First-principle calculations of dielectric tensor of monolayer ReS_2 . a, Hermitian part of the dielectric tensor. **b,** Hermitian components of the dielectric tensor after the diagonalization. **c,** Skew-Hermitian part of the dielectric tensor. **d,** Skew-Hermitian components of the dielectric tensor after the diagonalization. **e,** Three-dimensional view of principal optical axes variation for the Hermitian part of the dielectric tensor. **f,** Wavelength dependence of principal optical axes positions for the Hermitian part of the dielectric tensor in polar coordinates (see φ, θ in panel (e)). **g,** Three-dimensional view of principal optical axes variation for the skew-Hermitian part of the dielectric tensor. **f,** Wavelength dependence of principal optical axes positions for the skew-Hermitian part of the dielectric tensor in polar coordinates (see φ, θ in panel (g)).

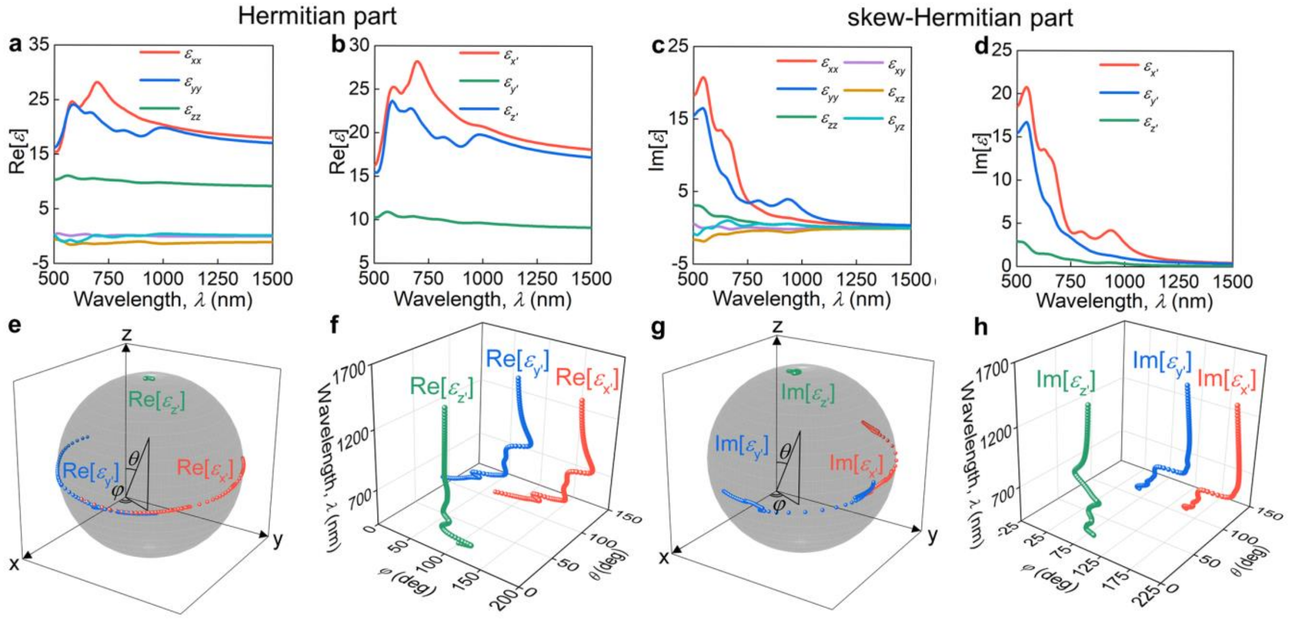

Supplementary Figure 20: First-principle calculations of dielectric tensor of bilayer ReS_2 . a, Hermitian part of the dielectric tensor. **b,** Hermitian components of the dielectric tensor after the diagonalization. **c,** Skew-Hermitian part of the dielectric tensor. **d,** Skew-Hermitian components of the dielectric tensor after the diagonalization . **e,** Three-dimensional view of principal optical axes variation for the Hermitian part of the dielectric tensor. **f,** Wavelength dependence of principal optical axes positions for the Hermitian part of the dielectric tensor in polar coordinates (see φ, θ in panel (e)). **g,** Three-dimensional view of principal optical axes variation for the skew-Hermitian part of the dielectric tensor. **f,** Wavelength dependence of principal optical axes positions for the skew-Hermitian part of the dielectric tensor in polar coordinates (see φ, θ in panel (g)).

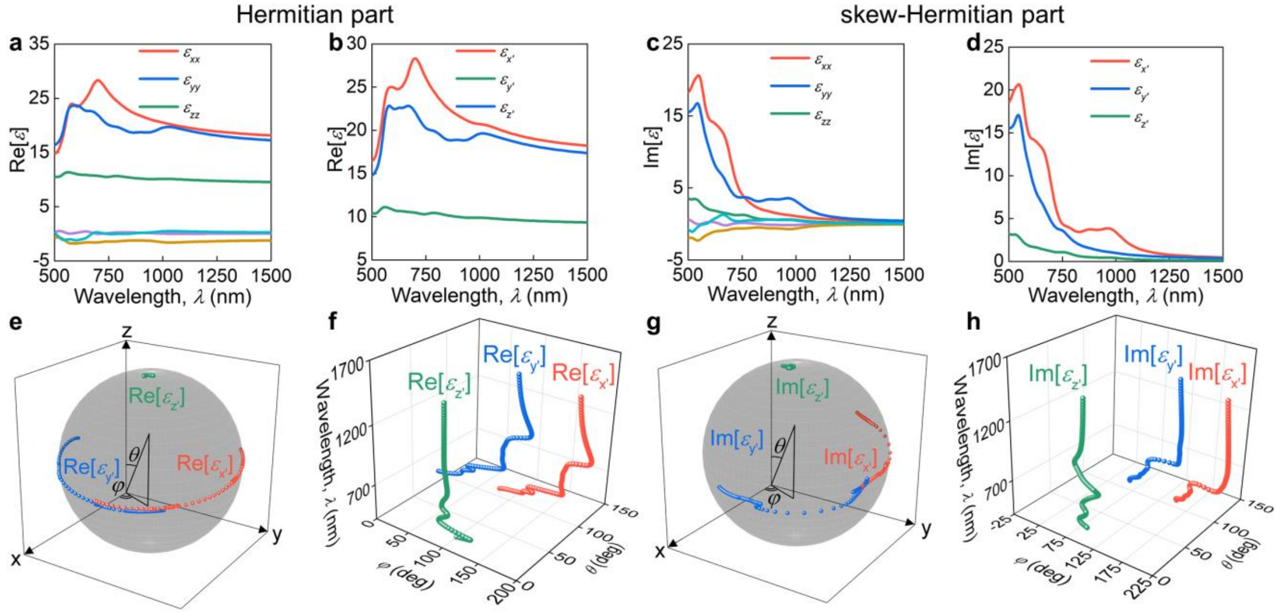

Supplementary Figure 21: First-principle calculations of dielectric tensor of trilayer ReS_2. a, Hermitian part of the dielectric tensor. **b,** Hermitian components of the dielectric tensor after the diagonalization. **c,** Skew-Hermitian part of the dielectric tensor. **d,** Skew-Hermitian components of the dielectric tensor after the diagonalization. **e,** Three-dimensional view of principal optical axes variation for the Hermitian part of the dielectric tensor. **f,** Wavelength dependence of principal optical axes positions for the Hermitian part of the dielectric tensor in polar coordinates (see φ, θ in panel (e)). **g,** Three-dimensional view of principal optical axes variation for the skew-Hermitian part of the dielectric tensor. **f,** Wavelength dependence of principal optical axes positions for the skew-Hermitian part of the dielectric tensor in polar coordinates (see φ, θ in panel (g)).

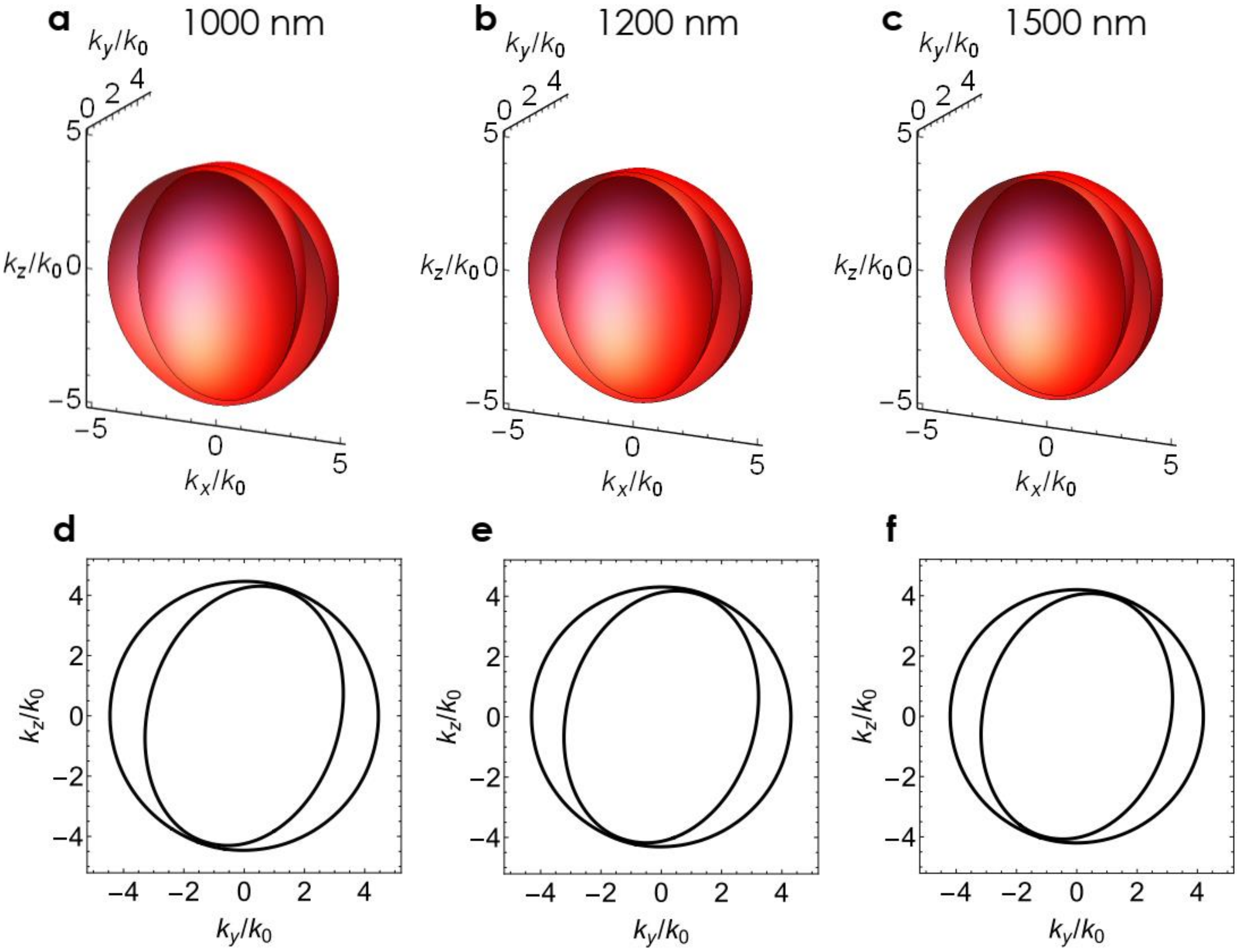

Supplementary Figure 22: Birefringent effect in ReS_2 under normal irradiation. a-c, Isofrequency surfaces of ReS_2 at a series of wavelengths. The surfaces are obtained by solving the characteristic Fresnel equation for the Hermitian part of the permittivity tensor as if the non-Hermitian part were absent. **d-f,** Corresponding cuts of the isofrequency surfaces by the *yz* plane revealing the slanted geometry.

SUPPLEMENTARY REFERENCES

1. Mooshammer, F. *et al.* In-plane anisotropy in biaxial ReS_2 crystals probed by nano-optical imaging of waveguide modes. *ACS Photonics* **9**, 443–451 (2022).

2. Munkhbat, B., Wróbel, P., Antosiewicz, T. J. & Shegai, T. O. Optical constants of several multilayer transition metal dichalcogenides measured by spectroscopic ellipsometry in the 300–1700 nm range: high index, anisotropy, and hyperbolicity. *ACS Photonics* **9**, 2398–2407 (2022).

3. Choi, Y. *et al.* Complete determination of the crystallographic orientation of ReX_2 (X = S, Se) by polarized Raman spectroscopy. *Nanoscale Horizons* **5**, 308–315 (2020).

4. Sun, L. *et al.* Spin-orbit splitting in single-layer MoS_2 revealed by triply resonant Raman scattering. *Phys. Rev. Lett.* **111**, 126801 (2013).

5. Lin, M.-L. *et al.* Understanding angle-resolved polarized Raman scattering from black phosphorus at normal and oblique laser incidences. *Sci. Bull.* **65**, 1894–1900 (2020).

6. Hu, F. *et al.* Imaging exciton-polariton transport in $MoSe_2$ waveguides. *Nat. Photonics* **11**,

356–360 (2017).

7. Zenin, V. A. *et al.* Boosting local field enhancement by on-chip nanofocusing and impedance-matched plasmonic antennas. *Nano Lett.* **15**, 8148–8154 (2015).

8. Ocelic, N., Huber, A. & Hillenbrand, R. Pseudoheterodyne detection for background-free near-field spectroscopy. *Appl. Phys. Lett.* **89**, (2006).

9. Bai, Q., Perrin, M., Sauvan, C., Hugonin, J.-P. & Lalanne, P. Efficient and intuitive method for the analysis of light scattering by a resonant nanostructure. *Opt. Express* **21**, 27371 (2013).

10. Fradkin, I. M., Dyakov, S. A. & Gippius, N. A. Fourier modal method for the description of nanoparticle lattices in the dipole approximation. *Phys. Rev. B* **99**, 075310 (2019).

11. Hu, D. *et al.* Probing optical anisotropy of nanometer-thin van der waals microcrystals by near-field imaging. *Nat. Commun.* **8**, 1471 (2017).

12. Ermolaev, G. A. *et al.* Giant optical anisotropy in transition metal dichalcogenides for next-generation photonics. *Nat. Commun.* **12**, 854 (2021).

13. Gehlmann, M. *et al.* Direct observation of the band gap transition in atomically thin ReS_2. *Nano Lett.* **17**, 5187–5192 (2017).

14. Tongay, S. *et al.* Monolayer behaviour in bulk ReS_2 due to electronic and vibrational decoupling. *Nat. Commun.* **5**, 3252 (2014).
